# Two-layer closure method using anchor pronged clips for endoscopic full-thickness resection

**DOI:** 10.1055/a-2764-4418

**Published:** 2026-02-05

**Authors:** Takashi Kodato, Yoichi Yamamoto, Masao Yoshida, Noboru Kawata, Kenichiro Furukawa, Etsuro Bando, Hiroyuki Ono

**Affiliations:** 1 38471 Division of Endoscopy, Shizuoka Cancer Center, Shizuoka, Japan; 2 Division of Gastric Surgery, Shizuoka Cancer Center, Shizuoka, Japan

**Fig. 1:**
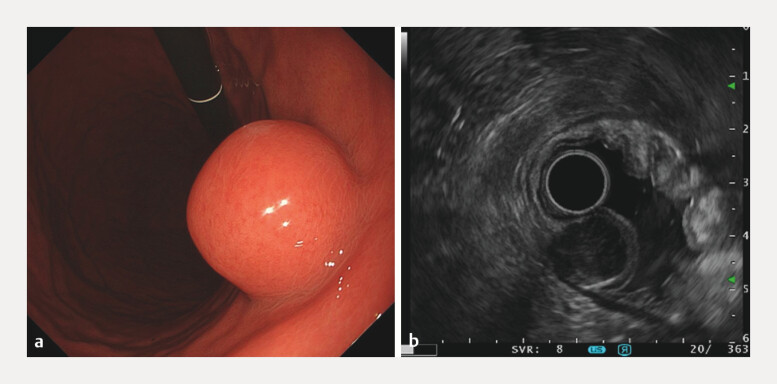
Esophagogastroduodenoscopy **a** and EUS **b** revealed a 25-mm submucosal tumor in the lower gastric body. EUS, endoscopic ultrasound.

**Fig. 2:**
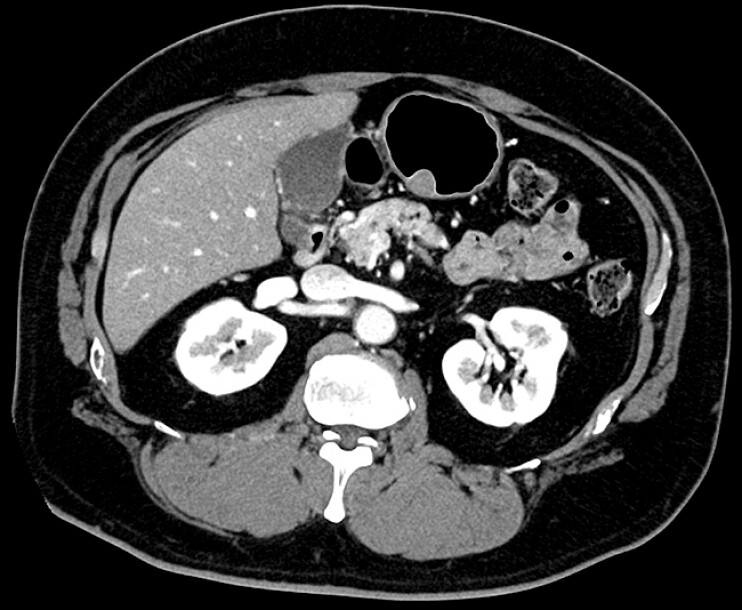
Computed tomography revealed a 25-mm gastric submucosal tumor and no evidence of metastasis.

A 54-year-old man underwent esophagogastroduodenoscopy, which revealed a 25 mm submucosal tumor in the lower gastric body ( Fig. 1 **a** ). Endoscopic ultrasound-guided fine-needle biopsy led to a diagnosis of gastric gastrointestinal stromal tumors (GISTs; Fig. 1 **b** ). Computed tomography revealed no evidence of metastasis ( Fig. 2 ). We therefore proceeded with endoscopic full-thickness resection (EFTR) to treat this submucosal tumor ( Video 1 ).

The defect after EFTR was closed with four MANTIS clips for the muscle layer and additional clips for the mucosa following successful specimen extraction. EFTR, endoscopic full-thickness resection.Video 1

**Fig. 3:**
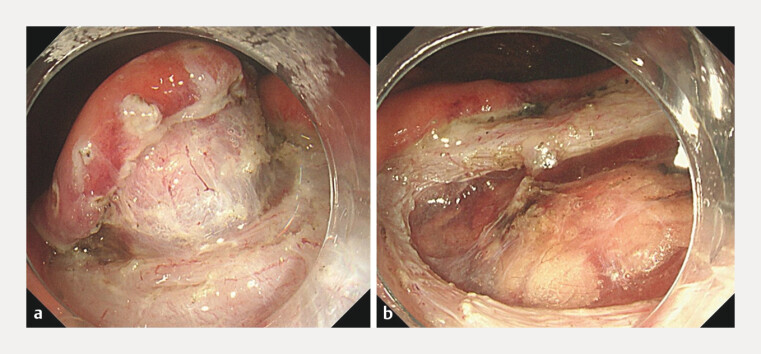
**a** The submucosa to minimize any resulting full-thickness defects prior to specimen removal. **b** Extraction of specimens with minimal muscle dissection.

Under general anesthesia, we carefully dissected the submucosa to minimize any resulting full-thickness defects prior to specimen removal ( Fig. 3 **a** ). The specimen was successfully extracted via a minimal muscle layer incision without capsule injury ( Fig. 3 **b** ).

**Fig. 4:**
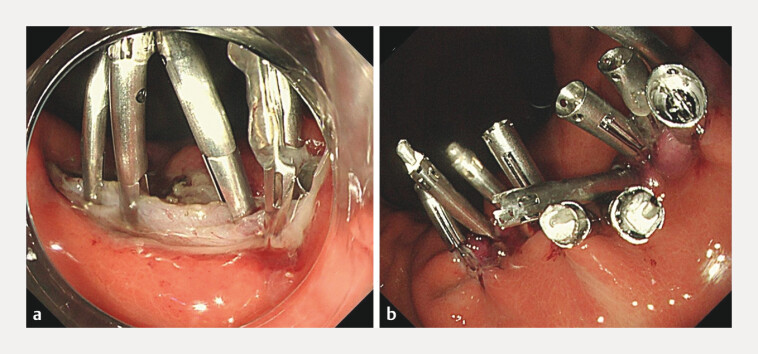
**a** Use anchor-pronged clips (MANTIS clips; Boston Scientific) to close the wound between the muscle layers. **b** Use MANTIS clips and conventional clips to close the mucosal defects.

Four anchor-pronged clips (MANTIS clips; Boston Scientific) were used to close the defect between the muscle layers of the stomach wall ( Fig. 4 **a** ). Subsequently, we used three additional MANTIS and five conventional clips to close the mucosal defects, achieving complete closure ( Fig. 4 **b** ).

**Fig. 5:**
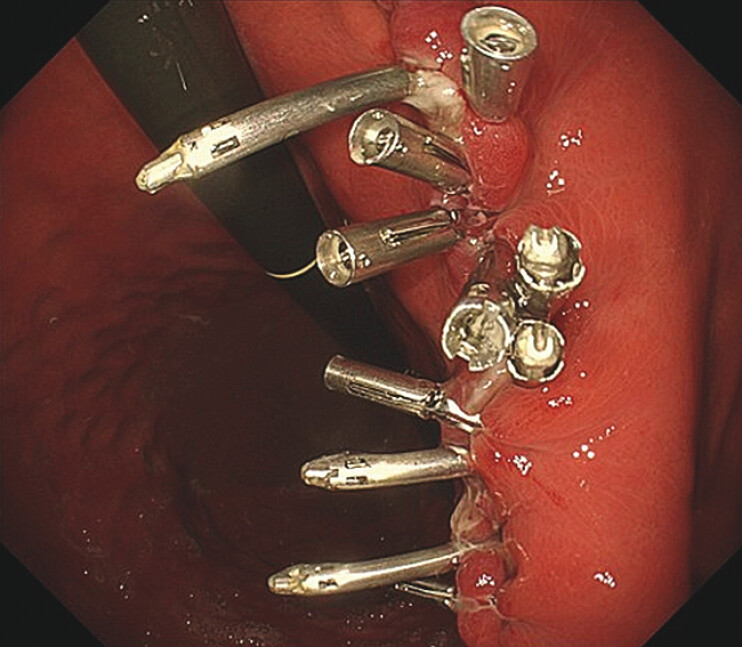
Postoperative day 3: complete closure of the treatment site was maintained.

Esophagogastroduodenoscopy on postoperative day 3 confirmed complete closure of the resection site ( Fig. 5 ). No postoperative complications such as bleeding or delayed perforation were observed, and the patient was discharged on postoperative day 6.

Postoperative histopathological evaluation confirmed negative horizontal and vertical margins, with a final diagnosis of very low risk GIST (Modified Fletcher Classification).

EFTR represents an effective treatment option for small GISTs 1 . However, delayed perforation at full-thickness closure sites after resection remains a major concern 2 .

While earlier methods focused on mucosal layer closure 1 , various techniques have since been proposed to improve closure security, averting perforation 3 4 . Our recommended approach employs MANTIS clips for the independent closure of the muscle and mucosal layers, similar to the standard two-layer surgical suturing technique 5 . This method offers more robust closure than mucosal-only suturing, and is therefore expected to reduce postoperative complications.

Endoscopy_UCTN_Code_TTT_1AO_2AO
